# Development of functional topography of the default mode subnetworks revealed by precision mapping

**DOI:** 10.1016/j.dcn.2026.101808

**Published:** 2026-08-29

**Authors:** Ying He, Jonah Kember, Hongxiu Jiang, Xiaoqian J. Chai

**Affiliations:** a State Key Laboratory of Cognitive Science and Mental Health, Institute of Psychology, Chinese Academy of Sciences, Beijing, 100101, China; b Department of Psychology, University of Chinese Academy of Sciences, Beijing, 100049, China; c Department of Neurology and Neurosurgery, McGill University, Montréal, QC, H3A 2B4, Canada

**Keywords:** Default mode subnetwork, Precision mapping, Memory, Functional topography

## Abstract

The default mode network (DMN) is a functionally and anatomically heterogeneous network comprising distinct subsystems that support internally directed processes such as memory and self-related thought. The development of functional brain networks is critical for cognitive maturation, yet how DMN subnetworks develop remains unclear. Group-average approaches obscure individual variability and blur spatial details, limiting precise characterization of network topography. Using precision mapping in 547 participants aged 5–21 years, we delineated individualized DMN subnetworks and examined their development across multiple aspects, including functional segregation and spatial topography. We found that DMN subnetworks become increasingly functionally segregated from each other and from non-DMN networks with age, and exhibit greater topographic distinctiveness through boundary sharpening and reduced spatial overlap. Furthermore, the memory-related subnetwork showed a negative association between age and spatial extent, with a smaller surface area associated with better episodic memory performance. Our findings underscore the importance of characterizing multidimensional functional and topographical features at the individual level to better understand typical neurodevelopment and neurodevelopmental and psychiatric conditions.

## Highlights

• We provide the first developmental characterization of default mode subnetworks using individual-level precision mapping.
• With age, subnetworks showed greater segregation and topographic distinctiveness, with sharper boundaries and less overlap.
• Memory-related subnetwork surface area decreases with age, and smaller area relates to better episodic memory.

## 1 Introduction

The default mode network (DMN) comprises interconnected and distributed brain regions and is thought to be involved in internal mental processes, such as remembering, thinking about future, and self/other-related thoughts(Randy L. Buckner, Andrews-Hanna, and Schacter, 2008; Menon, 2023). Disruptions of the DMN have been shown in many neuropsychiatric and developmental disorders(Anticevic et al., 2012; Konrad and Eickhoff, 2010; Padmanabhan, Lynch, Schaer, and Menon, 2017). Emerging evidence suggests that the DMN is a complex network that consists of distinct subnetworks, each supporting specific cognitive processes(Jessica R. Andrews-Hanna, Reidler, Sepulcre, Poulin, and Buckner, 2010; Braga and Buckner, 2017; Evan M. Gordon et al., 2020). During child development, the broader functional brain networks become increasingly segregated—characterized by stronger within-network and weaker between-network connectivity (Chai et al., 2014, Gu et al., 2015, Sherman et al., 2014). The DMN as a whole becomes more internally integrated while becoming increasingly segregated from other functional networks during development(Fan et al., 2021; Sun et al., 2025; C. Wang, Hu, Weng, Chen, and Liu, 2019). This growing segregation supports cognitive development by enabling more specialized network functioning(Baum et al., 2017; C. Wang et al., 2019). Previous studies suggested heterogeneous patterns of maturation across different regions of the DMN(Fan et al., 2021; Xiao, Zhai, Friederici, and Jia, 2016). However, how functional segregation among distinct DMN subnetworks develops during childhood remains poorly understood. One previous study examined DMN subsystem segregation but found no marked age-related changes in the neurotypical cohort. However, its relatively limited sample size and reliance on a small set of group-defined ROIs may have reduced its sensitivity to developmental changes and individual variation in DMN subnetwork organization(Bathelt and Geurts, 2021). Delineating the development of DMN subnetworks in a neurotypical population is essential, as it provides a crucial baseline for understanding alterations in related neuropsychiatric disorders.

To date, investigations of functional networks in children have largely relied on group-average (one-size-fits-all) parcellations. These group-average approaches introduce spatial blurring and fail to capture individual-specific features of brain organization. This limitation has motivated the growing use of precision mapping approaches, which identify functional networks at the individual level. By capturing individual variability, these methods offer improved reliability in linking brain organization to behavior and symptoms(Gratton et al., 2020; Kraus et al., 2023). Furthermore, inter-individual variability in functional brain networks may change across development(Demeter et al., 2025). In addition, studies using within-person precision mapping approaches have shown that the individual topography of functional networks — including their size, shape, and spatial location — differs markedly from the group average(Evan M. Gordon et al., 2017; Seitzman et al., 2019). These topographical variations become more refined during youth development and are associated with individual differences in cognition(Cui et al., 2020; Keller et al., 2023), with comparable alterations reported in clinical populations(Dickie et al., 2018; Lynch et al., 2024). Thus, in addition to assessing within- and between-network connectivity strength and other graph-based measures, this approach also enables the examination of individual topographic features and spatial extent of DMN subnetworks.

How to identify the DMN subnetworks remains an area of active research (R. L. Buckner and DiNicola, 2019; Smallwood et al., 2021). A commonly used approach is to divide it into three subnetworks, as proposed by Andrews-Hanna et al. (Jessica R. Andrews-Hanna et al., 2010; Andrews-Hanna, Smallwood, and Spreng, 2014), with similar parcellations also reported by Yeo et al. (Thomas Yeo et al., 2011). Based on these prior findings, we decided to parcellate the DMN into three subsystems, following Yeo et al. Previous task-fMRI studies have suggested distinct functional roles for DMN subnetworks. Midline core regions, broadly corresponding to DMNA, are engaged during self-referential judgments; the medial temporal lobe subsystem, broadly corresponding to DMNC, supports memory-based mental scene construction; and regions commonly assigned to DMNB, including the temporoparietal junction and dorsomedial prefrontal cortex, are involved in inferring others’ thoughts and personality traits (Jessica R. Andrews-Hanna et al., 2010; Mitchell, Neil Macrae, and Banaji, 2005; Saxe and Kanwisher, 2003). Consistent with these findings,a previous meta-analysis of the broad functional profiles of the three DMN subnetworks found that DMNA was more strongly associated with self-related processes, emotion and evaluation, as well as social and mnemonic processes shared with the other two subnetworks (J. R. Andrews-Hanna et al., 2014). DMNB was linked to mentalizing and social cognition, as well as story comprehension and semantic/conceptual processing. In contrast, DMNC showed strong associations with past and future autobiographical thought, episodic memory, and contextual retrieval. In the present cross-sectional study, we performed individual-level network parcellation in a cohort of 547 participants aged 5–21 years, including 538 participants aged 8–21 years who underwent 26 min of resting-state fMRI scanning and nine participants aged 5–7 years who underwent a shorter 21-minute protocol. Using the Yeo 17-network template as a reference, we identified three distinct DMN subnetworks (DMNA, DMNB, DMNC) based on individual functional connectivity profiles at the vertex level. To understand the development of these three DMN subnetworks, we first examined functional segregation of the three DMN subnetworks, quantified by the segregation index derived from the average within-network and between-network functional connectivity. This allowed us to assess whether the subnetworks became increasingly segregated from one another and from other non-DMN networks with age. Second, we mapped the topography of the three subnetworks individually and examined the topographical segregation, measured by boundary refinement and spatial differentiation between the subnetworks. Functional segregation does not capture the individualized spatial organization of functional networks, including variation in their locations and boundaries and in the spatial relationships between adjacent networks. Therefore, boundary sharpness and spatial overlap provide complementary measures of DMN subnetwork topography. Third, we examined age-related differences in the surface area of each subnetwork to characterize variation in their cortical representations across age. Finally, to explore the cognitive relevance of these changes, we focused on DMNC because, relative to DMNA and DMNB, its putative function is more specifically and consistently linked to episodic memory through medial temporal regions such as the parahippocampal cortex. We therefore tested whether DMNC metrics were associated with episodic memory performance.

## 2 Materials and methods

### 2.1 Data

All participants are from the Human Connectome Project in Development (HCP-D). HCP-D is a cross-sectional project including participants aged 5–21 years. HCP-D MRI data were acquired at four sites: Harvard University, University of California–Los Angeles, University of Minnesota, and Washington University in St. Louis(Somerville et al., 2018). Our analysis is based on Release 2.0 (N = 625). All participants provided informed assent or consent. Individuals exhibiting excessive head motion (frame-wise displacement > 0.25 mm for more than one-third of the scanning duration) were excluded from further analysis. The final sample consisted of 547 subjects aged 5–21 years (mean age = 15 ± 3.8 years, 285 females). The sample was predominantly White (63.4%), followed by individuals identifying as more than one race (15.8%), Black or African American (10.8%), Asian (7.5%), and Other (2.5%). The median annual household income of the sample was $119,000.

### 2.2 Image acquisition

MRI scans were acquired at 4 sites on 3 T Siemens Prisma scanners with a 32-channel Prisma head coil. T1w images were acquired with a 3D multi-echo MPRAGE (Magnetization Prepared Rapid Gradient Echo) sequence with an in-plane acceleration factor of 2. The scanning parameters included: repetition time of 2500 ms, echo times of 1.8, 3.6, 5.4, and 7.2 ms, inversion time of 1000 ms, flip angle of 8 °, 208 slices, and a voxel resolution of 0.8 mm isotropic. The fMRI scans are acquired with a 2D multiband gradient-recalled echo (GRE) echo-planar imaging (EPI) sequence (MB8, TR/TE = 800/37 ms, flip angle = 52) and 2.0 mm isotropic voxels covering the whole brain (72 oblique-axial slices). To mitigate bias associated with a specific phase-encoding direction, functional scans were acquired in pairs, with one run using anterior-to-posterior (AP) and the other using posterior-to-anterior (PA) phase encoding. Participants aged 8 years and older underwent 26 min of resting-state scanning, consisting of four runs of 6.5 min each. For younger participants aged 5–7 years (n = 9), a shorter protocol was used, comprising six runs of 3.5 min each, totaling 21 min.

### 2.3 Image preprocessing

In our study, we utilized preprocessed T1-weighted and fMRI images obtained from the NIMH Data Archive (NDA). The data were processed using the HCP-recommended preprocessing pipeline, *fMRIVolume*, which includes realignment, correction for spatial distortions, and registration of the functional images to the structural images with minimal smoothing (only due to interpolation, with no explicit smoothing). Artifact removal was performed using *sICA + FIX*, which regresses out motion- and artifact-related ICA components. Finally, *fMRISurface* was used to project gray matter voxels onto the cortical ribbon and register them to the fs_LR 32k surface space.

### 2.4 Precision mapping

We employed a template-matching approach similar to that used in previous studies(Hermosillo et al., 2024). Compared with other precision mapping methods—such as Infomap, non-negative matrix factorization, and Overlapping MultiNetwork Imaging mapping—template matching requires less scan time to obtain reliable personalized maps(Hermosillo et al., 2024). In this study, we used Yeo’s 17-network parcellation as the template. For each vertex, we computed its functional connectivity profile with all the other vertices and retained the top 5% of connections(Kember, He, Gracia-Tabuenca, Barnett, and Chai, 2026). We also examined the top 1% and 3% of connections and compared the consistency of parcellation across different thresholds using the Adjusted Rand Index, to ensure that the results were not driven by the specific choice of threshold. We then calculated the Dice similarity coefficient between each vertex’s connectivity profile and each of the 17 Yeo networks, generating a vertex-by-network similarity matrix. Each vertex was assigned to the network with which it showed the highest similarity. Subsequently, we applied the Connectome Workbench (https://www.humanconnectome.org/-

software/connectome-workbench) command to identify and remove small isolated clusters smaller than 40 mm^2^. These small clusters were then spatially reassigned to the nearest neighboring network to ensure spatial contiguity. Finally, we obtained the individual network parcellation for each subject. We conducted a split-half analysis to assess the reliability of individualized network maps (see Supplementary Materials for details).

### 2.5 Homogeneity of functional networks

To evaluate the effectiveness of our precision mapping approach, we calculated the within-network homogeneity, following procedures used in previous studies(E. M. Gordon et al., 2016; Kong et al., 2019). Specifically, we computed the Pearson correlation coefficients among all vertices within each network and then averaged these values across all networks for both the precision-mapped parcellation and the group-level Yeo's 17-network parcellation. A paired *t*-test was conducted to assess whether within-network homogeneity was significantly higher for individual-specific parcellations compared to the group-level parcellation.

### 2.6 Functional segregation of DMN subnetworks

To investigate the functional organization of DMN subnetworks, we calculated the functional segregation index, a metric that has been used in previous studies(Chan, Park, Savalia, Petersen, and Wig, 2014). We first extracted the time series of all vertices and computed pairwise Pearson correlations, resulting in a vertex-wise functional connectivity matrix. Next, we applied Fisher’s z-transformation to the connectivity values. For each network, we calculated the segregation index as the difference between its average within-network connectivity (FC_W_) and its average between-network connectivity (FC_B_), normalized by the within-network connectivity, as shown in the formula. We computed the within-DMN segregation (each DMN subnetwork vs. the other two DMN subnetworks) and whole-brain segregation (each DMN subnetwork vs. all 16 other networks).

$$\mathrm{Functional}\;\mathrm{segregation}=\;\frac{{\mathrm{FC}}_{W}-{\mathrm{FC}}_{B}}{{\mathrm{FC}}_{W}}$$

### 2.7 Density map of DMN subnetworks

To examine individual differences in the topography of DMN subnetworks, we computed the density map of the DMN subnetworks. The density map was generated by calculating the proportion of individuals for whom a given vertex belonged to a specific functional network. Higher values indicate that a greater proportion of participants assigned that vertex to the given network. To better visualize age-related changes in the spatial distribution of functional networks, subjects were divided into three age groups based on age: children (≤12 years, n = 147), adolescents (12–18 years, n = 262), and adults (≥18 years, n = 138). For each group, we generated density maps to visualize the spatial location of the network.

### 2.8 Sharpness of the boundary between DMN subnetworks

To quantify the topographical distinctiveness of DMN subnetworks, we calculated the boundary sharpness between adjacent networks. First, we used the Connectome Workbench command to identify spatially contiguous clusters within each functional network. Then, we generated borders outlining each cluster, and extracted the vertex indices corresponding to these borders. For each cluster, we computed its mean time series and identified nearby vertices within a 5 mm distance of each border vertex, adapted from our previous work(Kember et al., 2026). These nearby vertices were classified as either inside vertices (belonging to the same cluster) or outside vertices (belonging to different clusters). We then calculated the Pearson correlation between the cluster’s mean time series and the time series of each nearby border vertex. To assess boundary sharpness, we computed the absolute value of Cohen’s d for the difference in raw correlation values between inside and outside vertices. The outside vertices were further categorized into different functional networks based on individual-level precision mapping. This allowed us to compute boundary sharpness between each cluster and each adjacent functional network. If the number of outside vertices belonging to a given network was fewer than 5, the sharpness was not calculated for that network pair. Finally, we averaged the sharpness values of all clusters belonging to the same network to obtain network-to-network sharpness estimates. We separately computed the sharpness between each DMN subnetwork and the other two DMN subnetworks.

### 2.9 Spatial overlap between DMN subnetworks

To quantify spatial overlap between DMN subnetworks, we built upon the individualized precision mapping results. While precision mapping assigns each vertex exclusively to a single subnetwork through a winner-take-all procedure, functional overlap between subnetworks may exist in practice. To capture this potential overlap, we adopted the following approach. For each subnetwork, we first computed the mean BOLD time series by averaging all vertices within its subject-specific precision mapping-defined spatial extent. We then calculated Pearson correlation coefficients between this mean time series and every cortical vertex across the brain. The resulting correlation maps underwent z-score normalization to standardize the distribution. To allow vertices to be associated with multiple subnetworks based on their functional connectivity strength, each subnetwork was redefined as the set of vertices exceeding a functional-connectivity threshold, which we systematically varied from 0.0 to 2.0. Primary results are reported at a threshold of 1, with additional results provided in the Supplementary Materials. We selected this threshold because, after z-score normalization, a value of 0 corresponds to the mean correlation and 2 represents two standard deviations above the mean—indicative of high connectivity. Higher thresholds retain very few vertices and produce minimal overlap across regions. For each subnetwork pair, overlapping vertices satisfying the threshold criterion for both subnetworks were identified. We calculated the surface area of each cortical vertex to determine both the overlap area and the combined area of the two subnetworks. The overlap ratio, computed as the overlap area divided by the combined area, provided a normalized measure of spatial differentiation.

### 2.10 Surface area of DMN subnetworks

To quantify the spatial extent of each DMN subnetwork, we first calculated the surface area (in mm²) for each cortical vertex. The relative proportion of each network was then calculated by summing the surface areas of its constituent vertices and dividing by the total cortical surface area. After examining age effects on surface area proportion, we further examined age-related changes in the topography of DMN subnetworks. To characterize how the spatial distribution of individualized network parcellations changes with age, we compared individual maps with the Yeo template as a reference, which represents the canonical spatial distribution of each network. By comparing individual network maps against this reference on a vertex-by-vertex basis, we identified vertices that showed different subnetwork assignments across participants of different ages. For example, by comparing an individual's DMNC map against the group-level DMNA and DMNB maps, we could determine whether regions contributing to the smaller DMNC representation associated with greater age corresponded to canonical DMNA or DMNB territory in the reference map. The conceptual meaning, computational definition, and interpretation of the primary brain measures are summarized in Table 1.

**Table 1:** Summary of the Primary Brain Measures.

Measure	Conceptual meaning	Computational definition	Interpretation
Functional segregation	The overall extent to which connectivity within a network is stronger than its connectivity with other networks.	Functional segregation was calculated as (FCW - FCB) / FCW, where FCW is the mean within-network connectivity and FCB is the mean connectivity between the target network and the comparison networks.	Higher values indicate that vertices within the network are, on average, more strongly connected to one another relative to vertices in other networks, reflecting greater network segregation.
Boundary sharpness	The degree of functional differentiation across the boundary between adjacent networks.	For each individualized network cluster, boundary sharpness was calculated as the absolute Cohen's d between raw correlations for vertices located inside versus outside the boundary within 5 mm and then averaged across clusters within each network.	Higher values indicate a larger functional contrast across the network boundary and therefore a more clearly delineated boundary between adjacent networks.
Spatial overlap	The extent to which two subnetworks share cortical vertices that are strongly associated with both networks' representative time series.	For each individualized subnetwork, a whole-cortex correlation map was generated by correlating its mean time series with every cortical vertex. Spatial overlap was calculated as the surface area of vertices showing high correlations with both subnetworks divided by their combined area.	Lower values indicate fewer vertices shared by the two network maps and therefore greater spatial differentiation between the subnetworks.
Surface-area proportion	The relative amount of cortical territory assigned to an individualized subnetwork.	The surface areas of all vertices assigned to the subnetwork were summed and divided by the participant's total cortical surface area.	Higher values indicate that the subnetwork occupies a larger proportion of the participant's cortical surface.

### 2.11 Assessing age-related effects on brain measures

We employed a general linear model (GLM) to examine the relationship between age and brain measures, including functional segregation, boundary sharpness, spatial overlap and surface area of DMN subnetworks. In these models, age was treated as the independent variable, while the brain measures served as dependent variables. Sex and scanning sites were included as fixed effects. To account for multiple comparisons, we applied false discovery rate (FDR) correction to the p-values associated with the age variable. As a sensitivity analysis, we additionally included mean framewise displacement as a fixed effect and refitted the models using linear mixed-effects models, with family membership included as a random intercept to account for relatedness among participants from the same family. Based on the primary findings, we further evaluated sex moderation and nonlinear age associations for the main outcome measures. None of the age × sex interactions were significant (Table S1). For most measures, BIC provided no evidence that the quadratic model fitted better than the linear model. DMNC surface area was the only measure showing meaningful evidence in favor of the quadratic specification (Table S2).

### 2.12 Cognitive measurement

To explore the cognitive relevance of changes in DMN subnetwork organization, we focused on DMNC because its medial temporal components are most specifically linked to episodic memory relative to DMNA and DMNB. We therefore examined whether DMNC metrics were associated with performance on the Picture Sequence Memory (PSM) test, a measure of episodic memory for temporally ordered visual events(Dikmen et al., 2014). We expected the PSM scores to increase with age, and correlate with the memory-linked DMNC network. In the PSM task, a series of illustrated objects and activities were presented on an iPad in a specific order. The sequence length varied from 6 to 18 pictures depending on the participant's age. Participants were asked to recall the correct order of the pictures and received points for each correctly recalled adjacent pair. This test assesses the acquisition, storage, and effortful recall of newly learned information. Higher scores indicate better episodic memory performance relative to age-matched peers.

## 3 Results

### 3.1 DMN subnetworks show increasing functional segregation with age

**Fig. 1:**
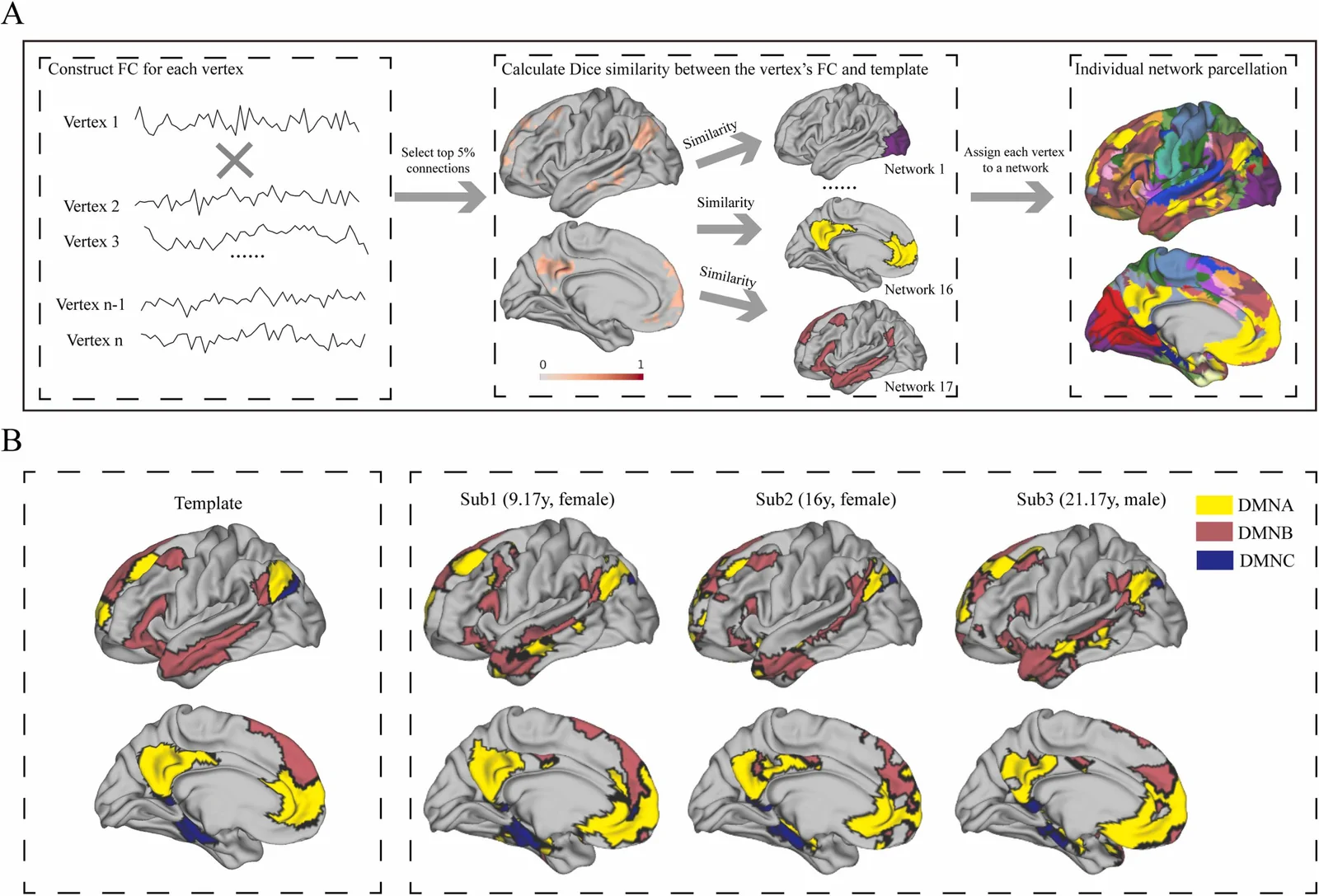
Overview of the precision mapping method. A. First, we constructed the functional connectivity profile for each vertex by calculating the Pearson correlation between that vertex and all other vertices. Next, we computed the Dice similarity metric to quantify the spatial similarity between each vertex’s connectivity profile and each of Yeo’s 17 networks. Finally, each vertex was assigned to the network with which it showed the highest similarity. B. The left panel shows the template of the DMN subnetworks based on the Yeo 17-network parcellation. The right panel displays three representative participants selected from different age groups, illustrating individual differences in the topographical distribution of the DMN subnetworks.

Here we employed a template-matching method to obtain individual parcellations of three DMN subnetworks (Fig. 1). After obtaining the individually mapped

functional networks, we first calculated the within-network homogeneity and compared it to that obtained using a group-level template. The homogeneity of individual networks was significantly higher than that of the group template (*t*_546_ = 15.42, *p* < 0.001), indicating the effectiveness of our precision mapping method. In addition, we computed the Adjusted Rand Index between each subject’s parcellation under a given threshold and the parcellations of the same and other subjects under different thresholds. We observed that parcellations from different thresholds within the same subject showed higher similarity than those between different subjects (Figure S1), suggesting that our precision mapping method produces consistent results within individuals and is robust to parameter variations.

**Fig. 2:**
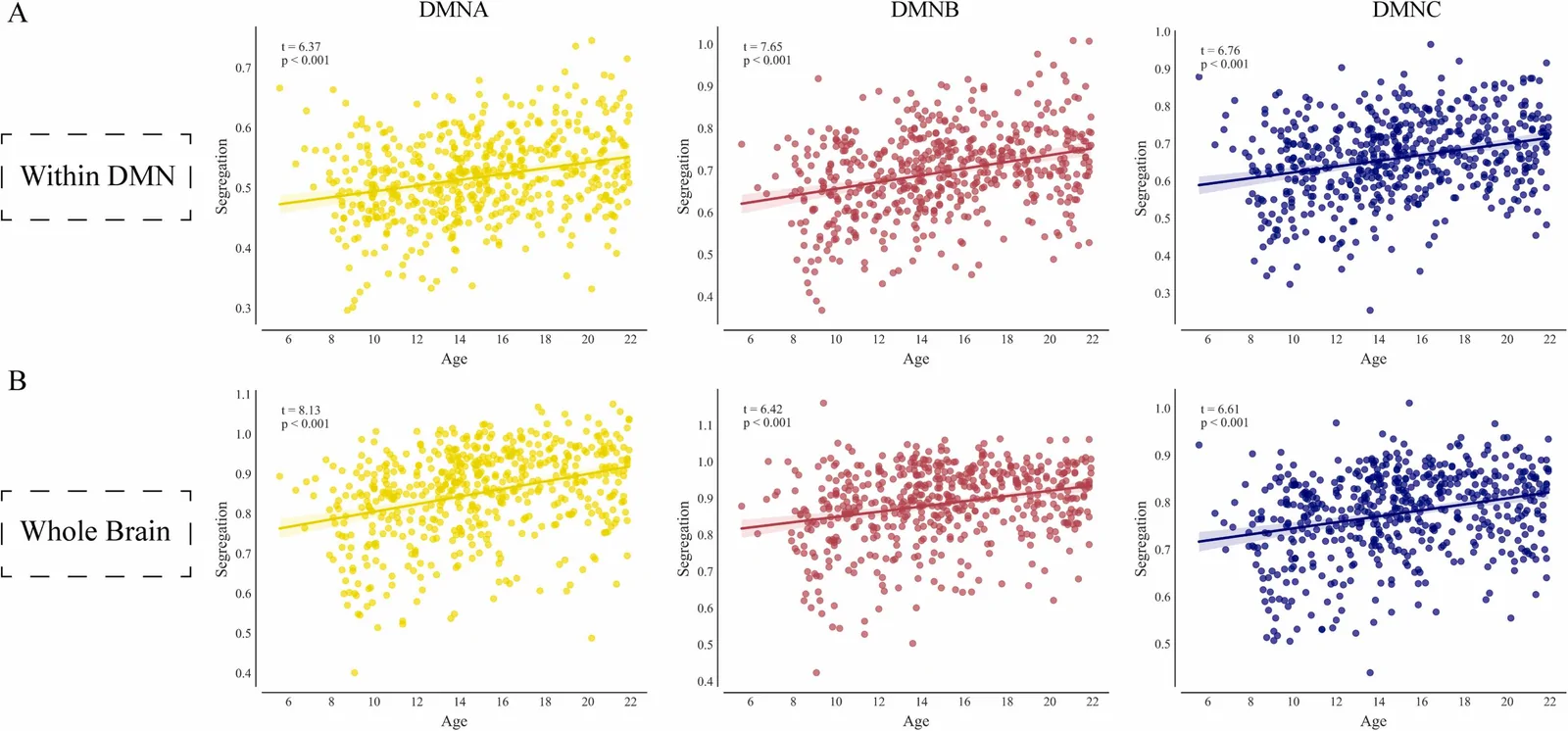
DMN subnetworks show increasing functional segregation with age. A. The segregation of each DMN subnetwork relative to other DMN subnetworks significantly increased with age. B. The segregation of each DMN subnetwork from all other large-scale functional networks also showed a significant age-related increase. Shaded bars reflect 95% confidence intervals of the regression line.

We quantified functional segregation by computing a segregation index based on within-network and between-network functional connectivity. Within the DMN, each subnetwork showed significantly increased functional segregation from the other two subnetworks with age (Fig. 2A, DMNA: *β* = 0.005, *t*_541_ = 6.37, *p*_corrected_ < 0.001; DMNB: *β* = 0.008, *t*_541_ = 7.65, *p*_corrected_ < 0.001; DMNC: *β* = 0.008, *t*_541_ = 6.76, *p*_corrected_ < 0.001). These findings demonstrate greater functional segregation among all three DMN subnetworks with increasing age.

In addition, we examined the age-related changes in functional segregation between each DMN subnetwork and the other 16 large-scale functional networks. Similar to segregation relative to other subnetworks of DMN, all DMN subnetworks showed significant increases in segregation from the other 16 networks with age (Fig. 2B, DMNA: *β* = 0.010, *t*_541_ = 8.13, *p*_corrected_ < 0.001; DMNB: *β* = 0.007, *t*_541_ = 6.42, *p*_corrected_ < 0.001; DMNC: *β* = 0.006, *t*_541_ = 6.61, *p*_corrected_ < 0.001). We also tested whether increasing functional segregation of DMNC from the other two DMN subnetworks and from all other 16 networks correlated with PSM scores, but no significant correlations were found (all *ps* ≥ 0.42). These findings further highlight a cross-sectional age-related pattern consistent with increasing functional differentiation of DMN subnetworks.

### 3.2 Topographical boundaries of DMN subnetworks become sharper with age

**Fig. 3:**
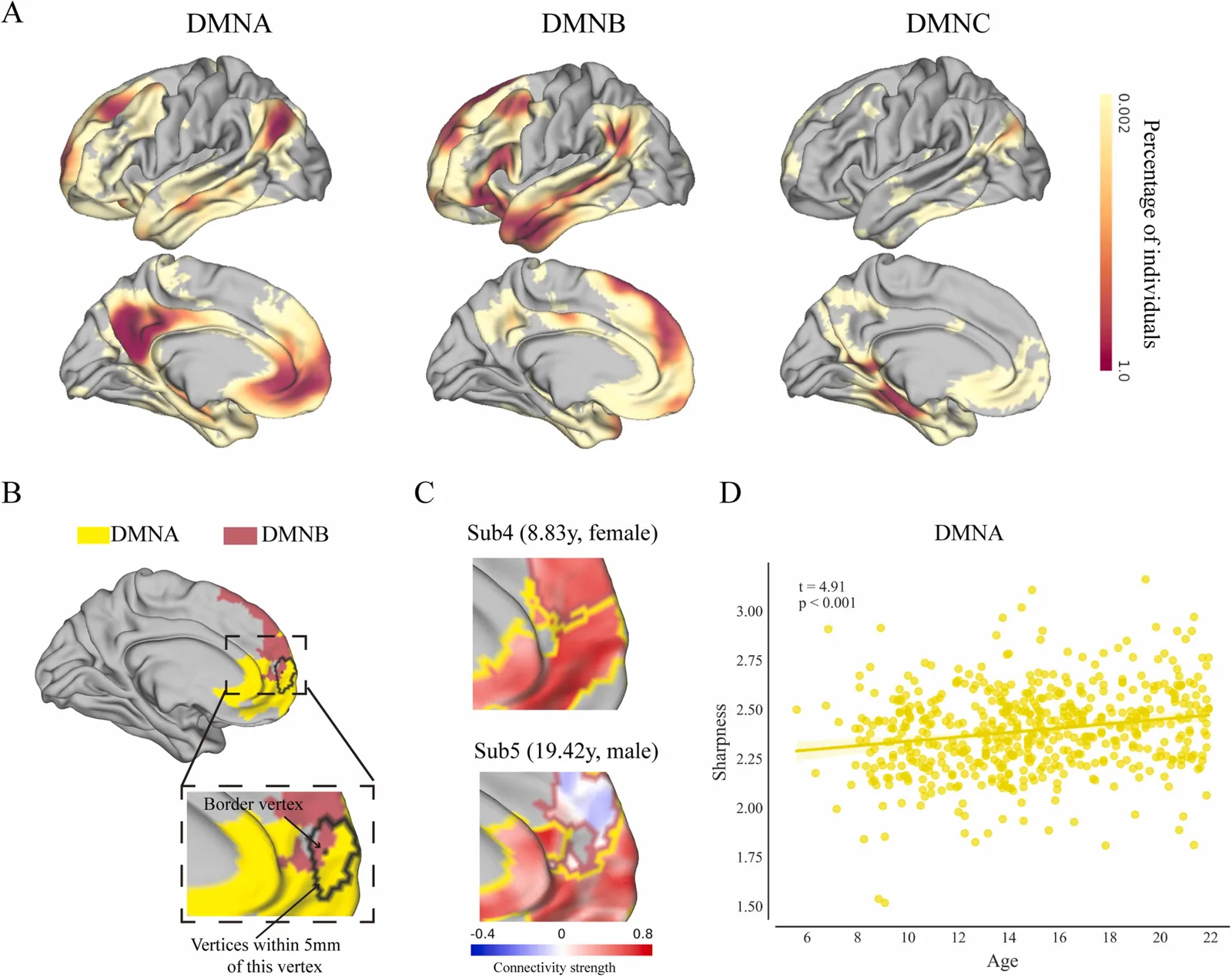
Topographical boundaries of DMN subnetworks became sharper with age. A. The density maps indicated the proportion of subjects for whom a given vertex was assigned to each of the three DMN subnetworks. The spatial locations of the three DMN subnetworks were primarily centered around their respective centroids within each cortical zone, but they also extended into regions typically associated with other subnetworks. B. Two clusters from different DMN subnetworks were selected as examples to illustrate how boundary sharpness was quantified. For each border vertex between the two clusters, we identified all vertices located within 5 mm and used them for the analysis. C. Two example subjects illustrating boundary sharpness between DMNA and DMNB. Red outline indicates the DMNB cluster, and the yellow outline indicates the DMNA cluster. The color bar represents the functional connectivity strength of each vertex to the mean time series of the DMNA cluster. The top panel shows a subject with a blurred boundary, where vertices along the border exhibit highly similar connectivity to both subnetworks. The bottom panel shows a subject with a sharper boundary, where border vertices display distinct connectivity profiles between the two subnetworks. Note that while most cluster vertices are shown for visualization, sharpness was calculated only for border vertices within 5 mm (panel B). D. The scatter plot shows that DMNA boundaries with both other subnetworks became significantly sharper with age.

Here, we examined the spatial distribution of the three DMN subnetworks among the participants using the density map (Fig. 3A). We found that the highest density regions of each subnetwork were primarily located within their respective canonical cortical zones, but also extended into regions typically associated with other subnetworks, albeit at lower densities. For instance, the posterior cingulate cortex and precuneus regions were typically assigned to DMNA, although some individuals showed assignments to DMNB. The anterior temporal regions were generally associated with DMNB, but a substantial proportion of participants had anterior temporal regions that were assigned to DMNA. Similarly, the ventral medial prefrontal cortex was most often assigned to DMNA, yet assignments to DMNB and DMNC were also observed in some individuals. This pattern reflects both individual variability and their shared characteristics, with the three networks being distributed yet adjacent.

Furthermore, we aimed to investigate whether the topographical boundaries between DMN subnetworks become sharper with age. To do so, we first identified boundary vertices based on their proximity (within 5 mm) to the borders between adjacent subnetwork clusters (Fig. 3B). Boundary sharpness was defined as the extent to which these vertices showed stronger functional connectivity with their own subnetwork compared to the neighboring one, indicating a clearer distinction between adjacent subnetworks. Here, we present two examples illustrating a blurred versus a sharp boundary (Fig. 3C). We examined age-related changes in mean boundary sharpness for each DMN subsystem, calculated by averaging the sharpness values of the boundaries between the given subnetwork and the other two subnetworks. DMNA showed a significant increase in mean boundary sharpness with age (*β* = 0.01, *t*_541_ = 4.91, *p*_corrected_ < 0.001, Fig. 3D), driven by sharpness increases at both its interfaces with DMNB (*β* = 0.01, *t*_541_ = 3.65, *p*_corrected_ < 0.001) and DMNC (*β* = 0.01, *t*_541_ = 2.78, *p*_corrected_ = 0.010, Figure S2). We didn’t find significant age-related changes in mean boundary sharpness in DMNB (*β* = 0.006, *t*_541_ = 1.77, *p*_corrected_ = 0.114) or DMNC (*β* = 0.002, *t*_541_ = 0.34, *p*_corrected_ = 0.731) after FDR correction. This pattern suggests that DMNA boundary sharpness increases with age, whereas DMNB and DMNC boundary sharpness show no significant association with age.

### 3.3 Spatial overlap of DMN subnetworks decreases with age

**Fig. 4:**
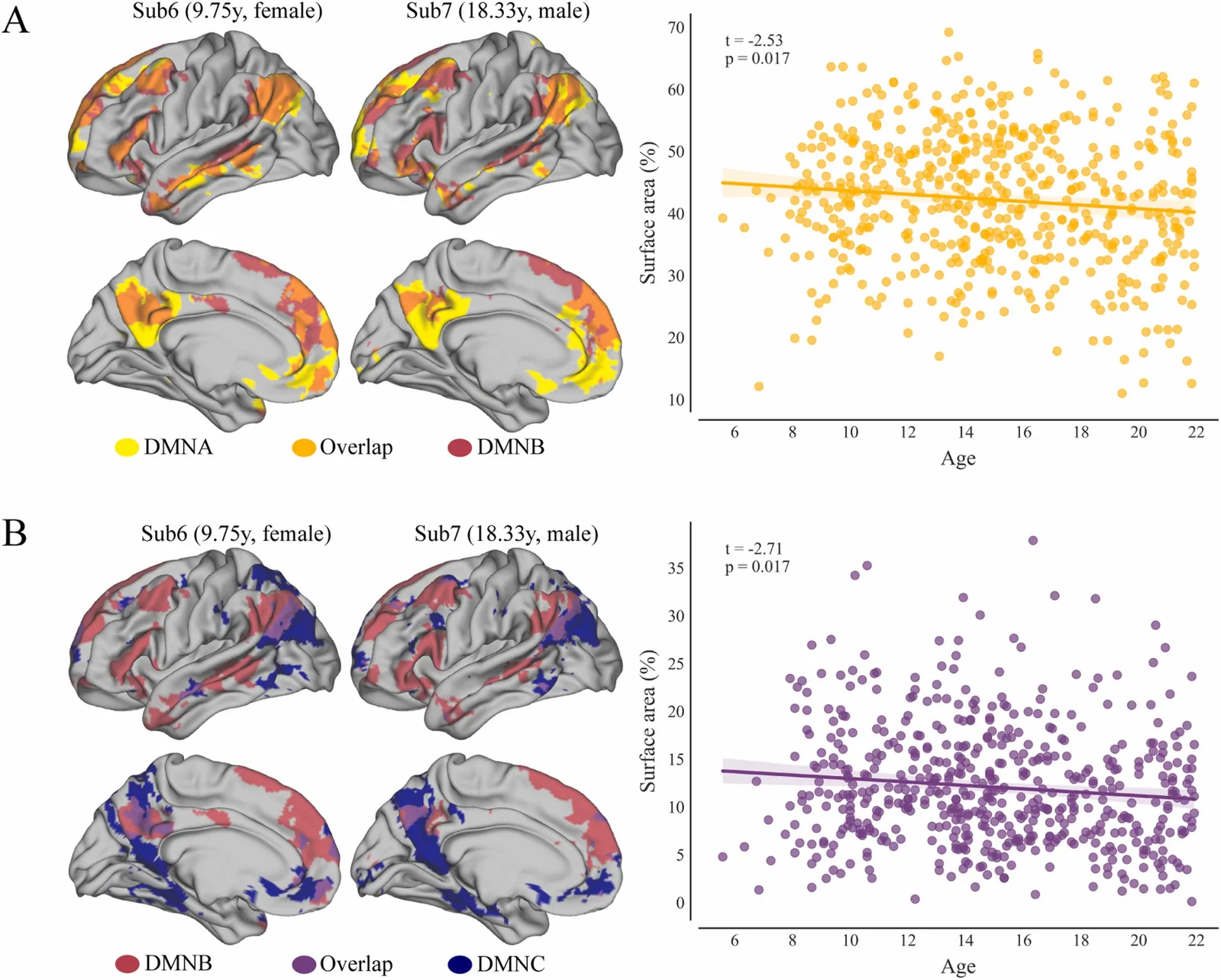
Overlap of DMN subnetworks decreased with age. A. Two examples illustrate the spatial overlap between DMNA and DMNB from a younger and an older participant, respectively. Only the left hemisphere is displayed for visual clarity. Analyses were conducted on both hemispheres. The accompanying scatter plot demonstrates a significant decrease in overlap with age. B. Two examples show the spatial overlap between DMNB and DMNC from a younger and an older participant. The scatter plot again indicates a significant age-related decline in overlap.

After demonstrating the refinement of boundaries with age, we next sought to investigate the spatial differentiation among DMN subnetworks. We defined spatial overlap between DMN subnetworks as vertices that simultaneously showed high functional connectivity with the mean time series of two subnetworks, where "high" was defined as connectivity exceeding the subject-specific mean by at least one standard deviation. We then examined how the surface area of these overlapping regions changed with age. Among the subnetworks, the spatial overlap between DMNA and DMNB exhibited the highest surface area proportion, and this overlap was significantly negatively correlated with age (*β* = −0.003, *t*_541_ = −2.53, *p*_corrected_ = 0.017, Fig. 4A). Similarly, the overlap between DMNB and DMNC also showed a significant negative correlation with age (*β* = −0.002, *t*_541_ = −2.71, *p*_corrected_ = 0.017, Fig. 4B). The spatial overlap between DMNA and DMNC did not show a significant relationship with age (*β* = 6.96E-05, *t*_541_ = 0.07, *p*_corrected_ = 0.947). Additional analyses across different correlation thresholds for defining high spatial overlap (from the subject-specific mean, to two standard deviations above) confirmed the robustness of the age-related decrease in overlap between DMNA and DMNB (Figure S3). These findings indicate greater spatial differentiation of DMN subnetworks with increasing age, particularly between DMNA and DMNB.

### 3.4 Surface area of DMNC decreases with age

**Fig. 5:**
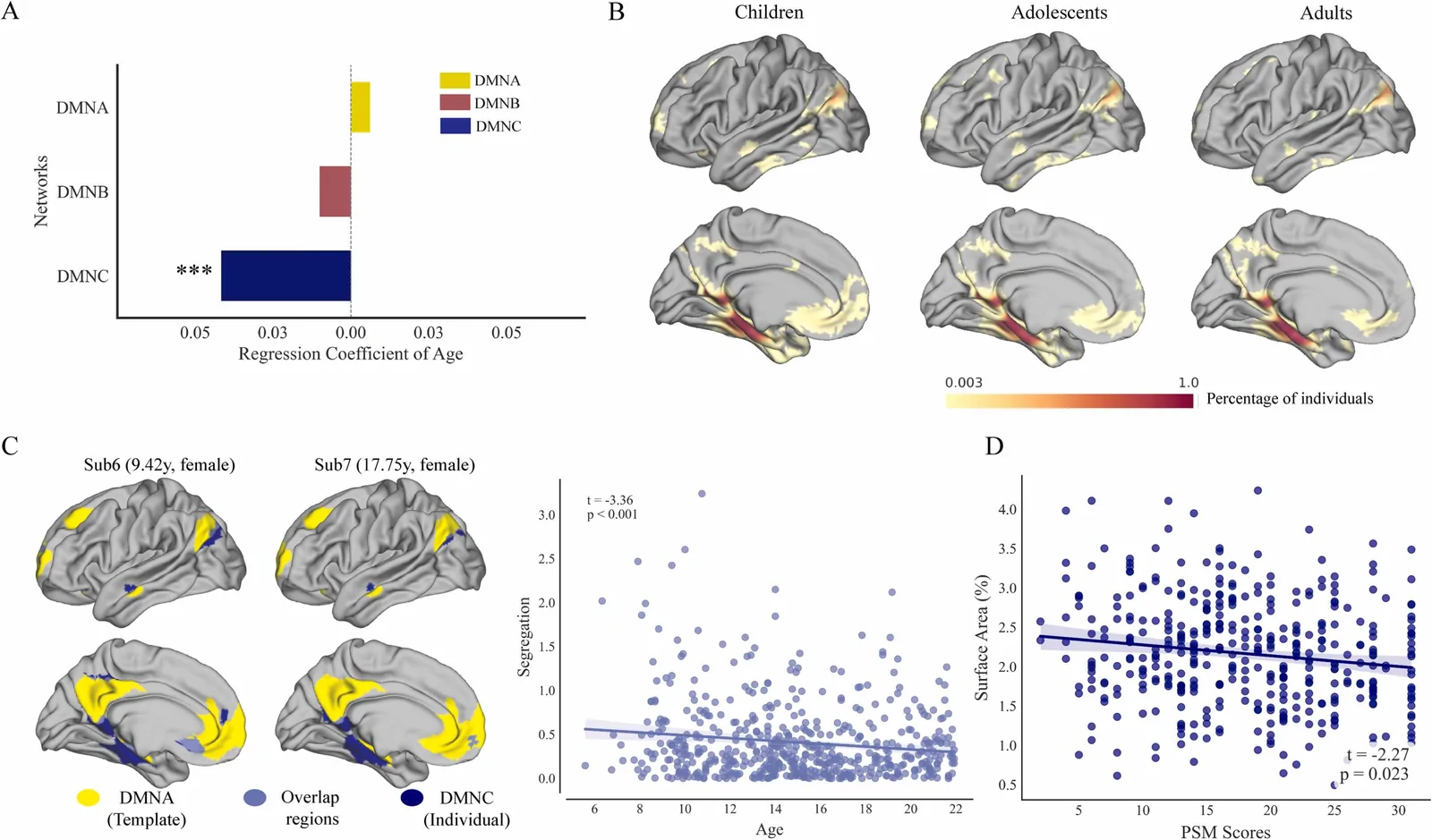
Surface area of DMNC decreased with age and memory performance. A. This bar plot illustrates the effect of age on the surface area of DMN subnetworks, based on a general linear model in which age was treated as the independent variable and surface area as the dependent variable, while controlling for confounding factors (sex, site, etc.). The width of each bar reflects the regression coefficient for age. Asterisks indicate levels of statistical significance after FDR correction: ***p < 0.001; **p < 0.01; *p < 0.05. B. Panel B shows the density maps for different age groups. Density values represent the proportion of subjects within each group in which a given vertex was assigned to the DMNC. C. The left panel shows two example subjects, illustrating the spatial overlap between individual DMNC (blue) and the group-level DMNA from template (yellow). The scatter plot demonstrates an age-related decrease in the overlap proportion (overlap area / individual's total DMNC area). D. A negative association was observed between the DMNC surface area proportion (individual’s DMNC area / individual’s total cortical area) and Picture Sequence Memory performances.

We first examined age-related changes in the surface area of DMN subnetworks using a GLM, controlling for sex and scanning sites. After correction for multiple comparisons, we observed a significant age-related decrease in the surface area of the DMNC subnetwork ( *β* = –0.04, *t*_541_ = –4.00, *p*_corrected_ < 0.001). No significant associations with age were found for the DMNA (*β* = 0.01, *t*_541_ = 0.58, *p*_corrected_ = 0.63) or DMNB (*β* = –0.01, *t*_541_ = –0.94, *p*_corrected_ = 0.45) subnetworks (Fig. 5A). To further illustrate age-related changes in DMNC surface area, subjects were divided into three age groups based on age: children (≤12 years, n = 147), adolescents (12–18 years, n = 262), and adults (≥18 years, n = 138). The corresponding density maps show that as age increases, the surface area of the DMNC gradually decreases and becomes more spatially constrained (Fig. 5B). A complementary quadratic analysis indicated that DMNC surface area was lower with increasing age, with a steeper age-related difference at younger ages and a flatter pattern at older ages (Figure S4). We also observed a significant age-related reduction of DMNC in the ventromedial prefrontal cortex (vmPFC), a region typically associated with the DMNA (Fig. 5B). This pattern indicates age-related differences in DMN subnetwork distribution, with smaller DMNC spatial extent associated primarily with canonical DMNA territory.

To further characterize the spatial organization of age-related changes in individualized DMNC, we compared each participant's DMNC map with the group-level template as a reference. This analysis allowed us to determine which canonical network labels corresponded to cortical locations contributing to the negative association between DMNC surface area and age. We first quantified the spatial overlap between each participant's DMNC and the group-level DMNA. As expected, the proportion of individual DMNC overlapping with group-level DMNA (overlap area / individual DMNC area) significantly decreased with age (β = –0.001, t_403_ = –3.36, p < 0.001, Fig. 5C). This finding indicates that increasing age was associated with less extension of individualized DMNC into cortical regions labeled as DMNA in the canonical parcellation. In contrast, when examining the overlap between youth DMNC and group-level DMNB, nearly all subjects showed minimal to no overlap (Figure S5). Thus, the cross-sectional age-related difference in DMNC spatial extent was localized primarily to canonical DMNA-associated cortical regions.

To examine the association between DMNC surface area and episodic memory, we also examined the relationship between PSM scores and the surface area of DMN subnetworks, controlling for age, sex, and scanning site. A significant positive association was observed between age and PSM score (*β* = 0.71, *t*_396_ = 8.05, *p* < 0.001), indicating that episodic memory performance improves with age. We found that the surface area of the DMNC subnetwork was significantly negatively correlated with PSM scores (*β* = −1.17, *t*_395_ = −2.27, *p* = 0.02), indicating that a reduced surface area was associated with better episodic memory performance. Together, these cross-sectional findings demonstrate that individual differences in DMNC spatial extent are associated with episodic memory performance (Fig. 5D).

### 3.5 Sensitivity analyses

To account for potential confounding by head motion and the non-independence of participants from the same family, we repeated the primary analyses using linear mixed-effects models. Age, sex, scanning site, and mean framewise displacement were included as fixed effects, while family membership was included as a random intercept. All previously observed associations between age and functional segregation remained significant. Whole brain segregation increased with age for DMNA (*β* = 0.0029, *z* = 4.63, *p*_corrected_ < 0.001), DMNB (*β* = 0.0020, *z* = 3.37, *p*_corrected_ = 0.001), and DMNC (*β* = 0.0017, *z* = 2.96, *p*_corrected_ = 0.003). Within-network segregation also increased with age for DMNA (*β* = 0.0020, *z* = 5.10, *p*_corrected_ < 0.001), DMNB (*β* = 0.0024, *z* = 4.30, *p*_corrected_ < 0.001), and DMNC (*β* = 0.0022, *z* = 3.27, *p*_corrected_ = 0.001). The positive association between age and DMNA boundary sharpness also remained significant (*β* = 0.0107, *z* = 4.07, *p*_corrected_ < 0.001). In addition, spatial overlap between DMNA and DMNB decreased significantly with age (*β* = −0.0051, *z* = −3.92, *p*_corrected_ < 0.001). For network surface area, DMNC showed a significant negative association with age (*β* = −0.0464, *z* = −3.82, *p*_corrected_ < 0.001). Finally, the hypothesized negative association between DMNC surface area and Picture Sequence Memory scores remained significant after adjustment for age, sex, scanning site, mean framewise displacement, and family structure (*β* = −1.176, *z* = −2.29, *p* = 0.022). Overall, these sensitivity analyses indicated that the primary findings were largely robust to additional adjustment for head motion and family relatedness.

## 4 Discussion

In this cross-sectional study, we systematically examined the age-related differences of segregation and specialization of DMN subnetworks in 547 participants aged 5–21 years, using precision mapping to obtain individualized parcellations of DMN subnetworks. Our findings reveal developmental segregation occurring across multiple dimensions. First, the three DMN subnetworks exhibited increased functional segregation from each other and from all other networks with age. Second, topographical segregation was observed: boundaries between DMNA and the other two subnetworks became significantly sharper with age, accompanied by reduced spatial overlap between DMNA and DMNB. Third, DMNC surface-area was negatively associated with age, and a smaller DMNC surface-area was associated with better episodic memory performance. Together, these findings provide a comprehensive characterization of age-related differences in DMN subnetwork organization, encompassing functional and topographical segregation and variation in spatial extent.

Here, we identified three distinct DMN subnetworks that varied across individuals and found that they became increasingly segregated from one another and from other functional networks with age. Previous studies have examined the functional segregation of the DMN by treating it as a single network. For example, one study reported increased within-network connectivity of the entire DMN during rest from childhood to adolescence(Fan et al., 2021). Similar effects have been observed in children during task states(C. Wang et al., 2019). More recently, a large-scale population study found that functional segregation of the canonical DMN, measured using the same index, increases rapidly with age and peaks at the end of the third decade(Sun et al., 2025). Our finding of increasing segregation among DMN subnetworks with age differs from that of Bathelt and Geurts (2021), who observed no marked age-related changes in DMN subsystem modularity in the neurotypical group. This discrepancy may partly reflect differences in sample size, network definition, and measurement. Specifically, their study assessed DMN subsystem organization using a relatively small set of group-defined regions, which may have reduced sensitivity to subtle and spatially distributed developmental changes in DMN subnetwork segregation. Our approach overcomes this limitation and reveals developmental effects showing that the DMN, a heterogeneous network as established by functional–anatomical evidence(Jessica R. Andrews-Hanna et al., 2010; J. R. Andrews-Hanna et al., 2014; Doucet et al., 2011; Greicius, Supekar, Menon, and Dougherty, 2009; Schmahmann et al., 2007; Thomas Yeo et al., 2011), becomes increasingly heterogeneous and functionally segregated over the course of development. Segregation of associative networks is consistently associated with enhanced cognitive performance, while the loss of such segregation in later life has been implicated in cognitive decline(Chan et al., 2014, Fair et al., 2007, Keller et al., 2023, Lopez et al., 2020, Ng et al., 2016, Sherman et al., 2014, Wang et al., 2019). DMN subnetworks share a common organizational motif that supports internally generated representations, with each network specialized for distinct processing domains(Buckner and DiNicola, 2019), and their segregation with development may reflect the enhancement of specific cognitive functions.

Our precision mapping results reveal individual parcellations with clear and specific spatial boundaries. Whereas functional segregation summarizes average within- and between-network connectivity, it does not capture individualized variation in network locations and boundaries or the spatial relationships between these adjacent networks. Boundary sharpness and spatial overlap provide additional information about these aspects of network topography. We examined the age-related effects on boundary sharpness between subnetworks and found that the boundaries around DMNA were significantly sharper with increasing age. Using the same measure in our previous work, we similarly observed age-related increases in boundary sharpness in the posterior subdivisions of the hippocampus(Kember et al., 2026). Although boundary sharpness was significantly correlated with functional segregation, the two measures were not equivalent, as reflected in their distinct developmental patterns. At the same time, we found that the spatial overlap between DMNA and DMNB decreases with age, suggesting that DMNA gradually emerges as a more spatially distinct and clearly delineated subnetwork. Notably, DMNA showed the most pronounced age-related changes in topographical segregation among all three DMN subnetworks. DMNA encompasses the so-called midline core regions of the DMN, including the anterior medial prefrontal cortex, angular gyrus, and posterior cingulate cortex, which are among the most consistently engaged regions within the network(Andrews-Hanna et al., 2010; Andrews-Hanna et al., 2014). Previous group-averaged studies examining functional connectivity between pairs of DMN nodes have reported age-related increases primarily in midline core regions, which is consistent with our findings(Fan et al., 2021). Convergent structural and functional connectivity analyses in prior work also suggest that PCC–mPFC connectivity (largely overlapping with DMNA) represents the most immature link within the DMN in children, further highlighting the pronounced age-related effects(Supekar et al., 2010). Importantly, previous studies have demonstrated that behavioral phenotypes across cognition, personality, and emotion can be predicted by individual-specific network topography with modest accuracy, comparable to predictions based on connectivity strength(Kong et al., 2019). Our study advances this line of research by introducing two novel measures of topographical segregation—boundary sharpness and spatial overlap—that uncover new insights into the developmental segregation of DMN subnetworks. The topographic characteristics of functional networks contribute to cognition and behavior(Smallwood et al., 2021; X. Wang et al., 2024). These topographical measures, enabled by within-person precision mapping, may provide new biomarkers for predicting behavioral outcomes and developmental trajectories at the individual level, complementing traditional connectivity-based approaches.

Finally, we examined age-related effects on the surface area of DMN subnetworks. While many studies have investigated developmental trajectories of total or regional cortical surface area based on group averages(Asato, Terwilliger, Woo, and Luna, 2010; Bethlehem et al., 2022; Vijayakumar et al., 2016), research focusing on the surface area of individual functional networks remains limited. One study on human lifespan functional connectome changes, using personalized mapping, reported a slight expansion of the whole DMN size during the first month of life but no significant change thereafter(Sun et al., 2025). This result highlights that treating the DMN as a single entity may obscure the heterogeneous developmental trajectories of its subnetworks. When we examined each subnetwork separately, we found that the surface area of DMNC decreased with age. The cortical locations contributing to this age-related difference corresponded predominantly to regions assigned to DMNA in the canonical parcellation, suggesting age-related variation in the spatial organization of DMN subnetworks. Critically, a smaller DMNC surface area was associated with better episodic memory performance, suggesting a link between its spatial organization and individual differences in episodic memory.

Our study has several limitations. First, we divided the DMN into three subnetworks; however, the exact number of DMN subnetworks remains unclear due to the complexity of DMN function, and our study does not address this question, which warrants further investigation. Second, when examining DMN-related cognitive performance—for example, the close association between the DMNA and self-related thoughts—we lacked corresponding behavioral measures to explore these relationships, which may require a broader behavioral phenotype. Third, our discussion of cognitive functions associated with DMN subnetworks primarily relies on previous literature rather than functional validation using specific tasks, which should be addressed in future studies. An important limitation of the present study is its cross-sectional design, which precludes the direct characterization of within-individual developmental changes and limits inferences about developmental trajectories. Future longitudinal studies with repeated assessments are needed to track how individualized functional networks reorganize over time and to determine whether changes in DMN subnetwork topography are associated with developmental changes in cognition and behavior.

In summary, our study establishes a novel, multi-dimensional framework for investigating age-related differences of DMN subsystem segregation by incorporating measures of functional segregation, topographical segregation, and spatial extent alterations through individualized precision mapping. We demonstrate that greater functional segregation of DMN subnetworks is observed alongside boundary sharpening and spatial differentiation—manifested as reduced overlap between subnetworks—as well as a smaller spatial extent of memory-related subnetwork that is associated with better episodic memory performance. By transcending group-averaged approaches and examining individual DMN subnetworks, our findings emphasize the importance of characterizing multi-dimensional functional and topographical features at the individual level to elucidate network segregation and specialization processes essential for typical neurodevelopment and relevant to neuropsychiatric and developmental disorders.

## Data and materials availability

Raw data are freely available through the Human Connectome Project, upon completion of a data-usage agreement: [https://nda.nih.gov/general-query.html?q=query=featured-datasets:HCP%20Aging%20and%20Development].

All the analysis and visualization code are available on GitHub: https://github.com/Chai-Neuro-Lab/Segregation-of-DMN-subnetworks

## Ethical statement

The authors assert that all procedures contributing to this work comply with the ethical standards of the relevant national and institutional committees on human experimentation and with the Helsinki Declaration of 1975, as revised in 2008.

## CRediT authorship contribution statement

**Xiaoqian J. Chai:** Writing – review & editing, Supervision, Investigation, Conceptualization. **Jonah Kember:** Writing – review & editing, Methodology, Investigation, Conceptualization. **Hongxiu Jiang:** Writing – review & editing, Investigation. **Ying He:** Writing – review & editing, Writing – original draft, Visualization, Methodology, Investigation, Conceptualization.

## Declaration of Competing Interest

The authors declare that they have no known competing financial interests or personal relationships that could have appeared to influence the work reported in this paper.

## Data Availability

Raw data are freely available through the Human Connectome Project. All the analysis and visualization code are available on GitHub: https://github.com/Chai-Neuro-Lab/Segregation-of-DMN-subnetworks

## Footnotes

Appendix A Supplementary data associated with this article can be found in the online version at doi:10.1016/j.dcn.2026.101808.
