# Supplementary material for: Development of functional topography of the default mode subnetworks revealed by precision mapping
Source: Dev Cogn Neurosci. 2026 Aug 29;81:101808. doi: 10.1016/j.dcn.2026.101808 (PMC13572014; doi:10.1016/j.dcn.2026.101808)
Supplement: Supplementary file 1 [file mmc1.docx]

Supplementary materials

Table S1. Sex moderation of age associations

| Measure class | Primary measure | Age*sex coefficient | *z* | *P_corrected_* |
| --- | --- | --- | --- | --- |
| Functional segregation | Whole-brain DMNA | -0.002 | -1.94 | 0.10 |
| Functional segregation | Whole-brain DMNB | -0.002 | -1.78 | 0.11 |
| Functional segregation | Whole-brain DMNC | -0.002 | -1.97 | 0.10 |
| Functional segregation | Within-network DMNA | 0.000 | -0.33 | 0.74 |
| Functional segregation | Within-network DMNB | -0.002 | -1.65 | 0.12 |
| Functional segregation | Within-network DMNC | -0.002 | -2.02 | 0.10 |
| Boundary sharpness | DMNA | -0.009 | -1.96 | 0.11 |
| Spatial overlap | DMNA*DMNB | 0.067 | 0.29 | 0.82 |
| Surface-area ratio | DMNC | -0.008 | -0.39 | 0.84 |

Table S2. Comparison of linear and quadratic models of age-related associations

| Measure class | Primary measure | Linear BIC | Quadratic BIC | $\Delta$BIC |
| --- | --- | --- | --- | --- |
| Functional segregation | Whole-brain DMNA | -1668.53 | -1665.78 | 2.75 |
| Functional segregation | Whole-brain DMNB | -1735.31 | -1730.70 | 4.61 |
| Functional segregation | Whole-brain DMNC | -1785.68 | -1782.08 | 3.60 |
| Functional segregation | Within-network DMNA | -2157.13 | -2152.64 | 4.49 |
| Functional segregation | Within-network DMNB | -1781.22 | -1774.92 | 6.30 |
| Functional segregation | Within-network DMNC | -1591.36 | -1585.36 | 6.00 |
| Boundary sharpness | DMNA | -99.40 | -93.13 | 6.26 |
| Spatial overlap | DMNA*DMNB | 4165.99 | 4165.19 | -0.80 |
| Surface-area ratio | DMNC | 1570.16 | 1566.36 | -3.80 |

Note. Lower BIC values indicate better model fit. ΔBIC was calculated as BIC_quadratic_ − BIC_linear_; therefore, negative values favor the quadratic model, whereas positive values favor the linear model.

Figure S1. **Threshold Robustness Analysis of Precision Mapping**. A Adjusted Rand Index (ARI) matrix comparing individual precision mappings at 95% versus 97% thresholds. Diagonal elements represent within-subject consistency across thresholds. B. ARI matrix comparing 95% versus 99% threshold mappings with identical diagonal interpretation.

Figure S2. **Age-related changes in boundary sharpness between each pair of DMN subnetworks.** The boundaries between DMNA and both other subnetworks became sharper with age. For DMNB, the boundary with DMNA became sharper, while the boundary with DMNC did not show a significant change. For DMNC, the boundary with DMNB became sharper, whereas the boundary with DMNA remained stable with age.

Figure S3. **Statistical Significance of Age-Overlap Surface Area Correlations Across Thresholds.** This figure illustrates the p-values of the correlation between overlap surface area and age across different z-score thresholds (ranging from 0 to 2). Each colored line represents the statistical significance of the relationship for different network overlaps: DMNA with DMNB (orange), DMNC with DMNA (blue), and DMNB with DMNC (purple). The gray dashed line indicates the conventional significance threshold of p = 0.05. The analysis demonstrates that the negative correlation between DMNA and DMNB overlap surface area and age is remarkably robust, maintaining statistical significance across all examined correlation thresholds.

Figure S4. **Nonlinear association between age and DMNC surface area.** The solid line represents the quadratic model fit, with the shaded area indicating the 95% confidence interval. Individual participants are represented by dots.

Figure S5. **Spatial overlap between individualized DMNC and group-level DMN subnetworks.** Histograms show the proportion of each participant’s individualized DMNC surface area overlapping with group-level DMNA and DMNB in the canonical template. The x-axis represents the spatial overlap proportion, and the y-axis represents the number of participants exhibiting each overlap proportion.

**Split-half reliability analysis**. To assess the split-half reliability of the personalized network maps, we randomly selected 109 participants from the full HCP-D sample using stratified sampling based on age group and acquisition site, with sex and motion bins additionally balanced during sampling. The sampled participants had a mean age of 14.72 years (SD = 3.99; range = 5.58-21.92 years) and mean FD of 0.143 mm (SD = 0.051). The sample included 61 females and 48 males. For each participant, the full resting-state time series was divided into two equal temporal halves. Personalized network maps were then generated independently for each half using the same template matching procedure. Split-half reliability was quantified using normalized mutual information (NMI) between the two half-specific network label maps. NMI measures the similarity between two parcellation label assignments while accounting for the entropy of each map, with higher values indicating greater correspondence. In addition, Dice similarity was calculated separately for the three default mode subnetworks, DMN-C, DMN-A, and DMN-B, to evaluate subnetwork-specific spatial overlap. Statistical significance was assessed using 1000 permutations in which Half 2 maps were shuffled across participants. For each permutation, we recalculated the mean mismatched Half 1-Half 2 similarity to form a between-subject null distribution. Empirical p values were computed as the proportion of null means greater than or equal to the observed within-subject mean, with a +1 correction.

The personalized network maps showed significant split-half reliability (Figure S6). The mean within-subject NMI was 0.669 (SD = 0.046), significantly higher than the permutation null distribution (null mean = 0.503, empirical *p* = 0.001). Subnetwork-specific Dice similarity was also significant for all three DMN subnetworks: DMN-C, 0.721 (SD = 0.099; null mean = 0.562, *p* = 0.001); DMN-A, 0.772 (SD = 0.053; null mean = 0.566, *p* = 0.001); and DMN-B, 0.814 (SD = 0.054; null mean = 0.622, *p* = 0.001).

Figure S6. Split-half reliability of individualized network. A Split-half reliability of individualized parcellations measured by normalized mutual information (NMI). B Split-half Dice similarity for the three individualized DMN subnetworks. Gray histograms show the permutation-based between-subject null distributions, and colored histograms show the observed within-subject split-half similarities. Dashed lines indicate null means, and solid lines indicate within-subject means. Empirical significance was assessed with 1000 permutations.
